# Effect of thalidomide on the proliferation of hepatoma cells assessed by osteopontin levels in nude mice

**DOI:** 10.3892/etm.2013.1010

**Published:** 2013-03-15

**Authors:** FAN LIN, JIE CAO, ZHIMING HUANG, ZHENGHAO PEI, WEILI GU, SHAOFENG FAN, KUNPING LI, JIEFENG WENG

**Affiliations:** 1 Department of General Surgery, The First People’s Hospital Affiliated to Guangzhou Medical University, Guangzhou, Guangdong 510180;; 2 Department of General Surgery, Guangdong General Hospital, Guangzhou, Guangdong 510030, P.R. China

**Keywords:** osteopontin, thalidomide, hepatocellular carcinoma

## Abstract

The aim of the present study was to investigate the inhibitory effects of thalidomide in the hepatocellular carcinoma nude mouse model in order to provide new insights into a comprehensive clinical intervention for hepatocellular carcinoma. MHCC97 cells were routinely cultured, passaged and adjusted to a single cell suspension with a concentration of 2×10^7^/ml. Six-week-old, BALB/C male nude mice were anesthetized and fixed in the prone position, then a subcapsular injection of the single cell suspension was administered into the spleen and their abdomens were closed. A laparotomy and left hepatic lobectomy was performed 14 days later and the abdomens were closed once again. Subsequent to the establishment of the hepatocellular carcinoma model, the nude mice were randomly divided into three groups, each consisting of 12 mice. The early intervention group were immediately provided with the post-operative thalidomide intervention, the late intervention group were provided with the post-operative thalidomide intervention one week subsequent to the surgery, and the negative control group were provided with a placebo intervention (0.9% physiological saline). Each intervention was continuously administered once per day for one week. The osteopontin (OPN) content of the liver tumors was detected using immunohistochemistry. The data were analyzed using an analysis of variance (ANOVA) test. There were significant differences in the OPN levels of the tumors among the early intervention, late intervention and negative control groups. Thalidomide may inhibit the generation of OPN and thereby inhibit the infiltration and metastasis of tumors; the immediate use of thalidomide following hepatectomy in the present study may block the invasion and metasis for liver cancer more effectively.

## Introduction

Cancer invasion and metastasis are difficult problems to overcome in cancer intervention. Genomics and transcriptional technology have been used to study the resected liver specimens of patients with hepatocellular carcinoma, as well as the molecular genetic features and gene expression profile in nude mouse and cell models of metastatic human hepatocellular carcinoma. It was identified that changes to the genes associated with liver metastasis occurred in the primary tumor stage and confirmed that osteopontin (OPN) had a significant predictive value and that it was the key transfer factor in hepatocellular carcinoma (1,2). This provided a new basis for the early diagnosis of hepatocellular carcinoma and for post-operative non-surgical intervention. These studies primarily answered the question of what invasion and metastasis of hepatocellular carcinoma are, but there have been few clinical studies concerning drug intervention in the invasion and metastasis of hepatocellular carcinoma. The present study aimed to evaluate whether thalidomide was able to inhibit the invasion and metastasis of hepatocellular carcinoma.

Thalidomide was first widely popularized and applied in West Germany in 1953 as a non-barbiturate sedative-hypnotic, mainly for the prevention of morning sickness. However, due to the teratogenic events (the side-effects were confined to pregnant women) associated with the drug, its use was forbidden in 1961. In 1991, D’Amato *et al*(3) identified that the teratogenic effect of thalidomide was related to the inhibition of new blood vessel formation. Subsequently, thalidomide was once more a focus of attention due to its effects in certain malignant tumors, particularly multiple myelomas. Thalidomide has also been identified to have extensive immune regulatory and anti-angiogenic effects. In 1998, thalidomide was approved by the FDA for use in clinical trials. There have been worldwide studies on thalidomide intervention in malignant tumors, however this cheap and well-known drug has commonly been limited in its use due to its unknown mechanism of action and the lack of support from evidence-based medical studies (4–7).

Thalidomide may act via a series of cascading effects with OPN involving new cell signaling pathways or media to control the expression and molecular behavior of intercellular substances in the hepatocellular carcinoma tumor microenvironment, and thereby directly or indirectly repress the invasion and metastasis of hepatocellular carcinoma. The elucidation of its mechanism may facilitate significant improvements in structure-activity studies of thalidomide and promote its use in tumor translational medicine.

## Materials and methods

### General data

A total of 36 BALB/C male nude mice (aged 6–7 weeks old), were purchased from the Laboratory Animal Center of the Medical College of Guangzhou Medical University. The MHCC97 cells were purchased from Guangzhou RiboBio Co., Ltd. (Guangzhou, China). The present study was carried out in strict accordance with the recommendations in the National Institutes of Health Guide for the Care and Use of Laboratory Animals. The protocol for animal use was reviewed and approved by the Institutional Animal Care and Use Committee (IACUC) of the First People’s Hospital Affiliated to Guangzhou Medical University (Guangzhou, China).

### Establishment of the nude mouse model of hepatocellular carcinoma

Following successful anesthesia, the nude mice were fixed in the prone position and conventional skin disinfection was performed. A 1-cm straight incision was made next to the spine 0.5 cm from the left costal margin, descending layer by layer into the abdomen. The incision was bluntly drawn to expose the spleen and then 0.1 ml MHCC97 single cell suspension was drawn up into a 1-ml insulin syringe and injected 0.3–0.4 cm into the spleen. Subsequent to being injected with 0.02 ml (∼8×10^6^ cells per mouse), the syringe was quickly withdrawn. The conditions of the mice were observed, including whether or not there was bleeding or intestinal oppression in the spleen and whether there was any contortion in the intestinal tract or mesentery. The abdomen was then closed layer by layer using a needle and 5-0 sutures.

### Partial hepatectomy and post-operative management of the nude mice

A partial hepatectomy of the liver was performed 14 days after splenic subcapsular inoculation with the MHCC97 cells. The nude mice were fed separately following the hepatectomy. The number of mice in each cage was also reduced as far as possible, generally to two mice in each cage. At one week post-hepatectomy, the mice were already being fed with sterile solid food, thereafter they were fed by injections of sterile water or physiological saline.

### Animal groups and specimens

Following the establishment of the hepatocellular carcinoma model, the nude mice were randomly divided into three groups, each consisting of 12 mice. Subsequent to the partial hepatectomy, the early intervention group were administered 5 mg/kg thalidomide as an immediate post-operative intervention, with continuous administration for one week. Specifically, the post-operatively anesthetized mice were fixed in an injection frame and 0.1 ml (150 mg/ml) thalidomide was injected into the tail vein with a 1-ml syringe. The late intervention group were treated with 0.1 ml (150 mg/ml) thalidomide via injection into the tail vein as a post-operative intervention one week after the surgery. They were also provided with continuous administration for one week. Following the partial hepatectomy, the negative control group were immediately injected with a placebo of 0.1 ml 0.9% physiological saline into the tail vein as the replacement intervention. Continuous administration was also provided for one week. The mice were sacrificed immediately prior to dissection. The liver tumor tissues were dissected, separated and fixed in formalin 21 days after the partial hepatectomy. The OPN contents of the liver tumors and paracarcinomatous tissues were detected using immunohistochemistry.

### Immunohistochemistry

Conventional perfusion fixation with 4% paraformaldehyde was performed on the mice. Samples were cut into slices. The primary antibody (rabbit anti-OPN antibody) was diluted with serum diluent (bovine serum albumin 1.00 g, 0.01 M PBS 100 ml and hydraulic nano 0.08 g) and added to the slices, which were kept at 4°C for 24–48 h. Subsequent to blotting the antibody, the slices were washed three times with 0.01 M KPBS for 5 min. In each group there were negative control groups, which included non-diluted primary antibodies (rabbit anti-OPN antibodies), secondary antibodies diluted with 0.01 M KPBS (anti-rabbit antibodies) and antibodies containing ABC or other complexes. The slices were incubated at room temperature for 2 h, washed three times with 0.01 M KPBS for 5 min and then rapidly washed three times with distilled water. DAB chromogen liquid was added to aid immunohistochemical analysis. Following dehydration with gradient alcohol, the slices were made transparent, sealed and images were captured.

### Calculation of results

The results of the staining process were judged using Bresalier semi-quantitative formulae. A total of 10 fields of view were randomly selected in each slice using high magnification (×200) and the double-blind method. The fields of view were divided into four grades according to the cell staining intensity and then scored as follows. Negative cells (-) showed no coloration (0 point); weakly positive cells (+) were light yellow (1 point); moderately positive cells (++) were claybank in color (2 points); and strongly positive cells (+++) were brown (3 points). The number of fields of view of each strength were counted and the average stain strength of each slice was calculated according to the following formula: IS (intensity score) = Σ [(0 × F0) + (1 × F1) + (2 × F2) + (3 × F 3)], F = % × 10 fields of view.

### Statistical analysis

Statistical analyses were performed using SPSS version 14.0 (SPSS, Inc., Chicago, IL, USA). The data are presented as mean ± SD and a t-test and one-way analysis of variance (ANOVA) was used for the comparison of the data between the groups. P<0.0091 was considered to indicate a statistically significant result.

## Results

### HE staining

**Figure 1:**
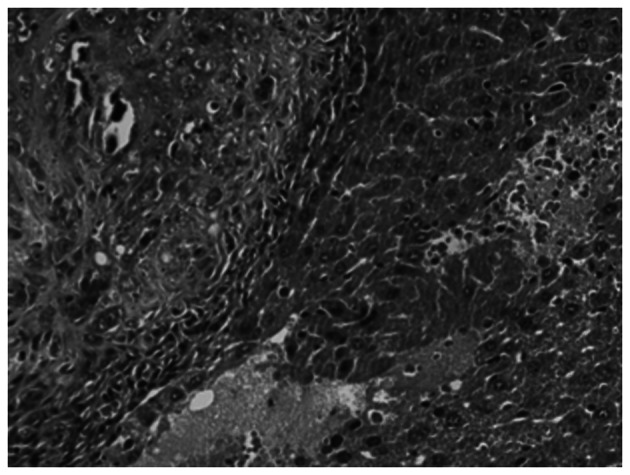
Hepatoma cells (magnification, ×100).

Yellow-white nodules were present in the livers of all 36 experimental animals that underwent partial hepatectomy subsequent to being sacrificed. The specimens of liver tumor tissue in all 36 cases were identified to be hepatic carcinoma following HE staining (Fig. 1).

### Immunohistochemistry results

**Figure 2:**
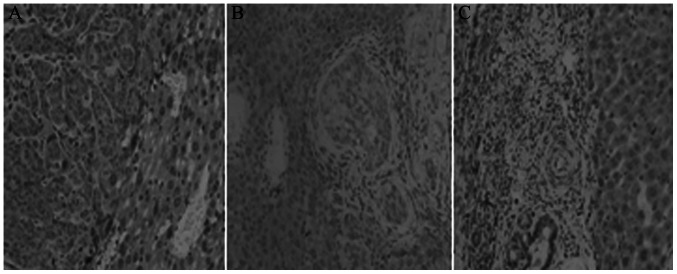
Immunohistochemistry results (magnification, ×100). (A) Early intervention group; (B) late intervention group; (C) negative control group.

As shown in Fig. 2, the OPN-positive cells in the tumor tissues of the early intervention group were fewer in number than those in the pericarcinomatous tissues. However, the OPN content in the pericarcinomatous liver cells in the late intervention group was lower than that in the tumor tissues.

### Immunohistochemistry results (qualitative determination)

The OPN level in the tumor tissues of the early intervention group (1.079±0.345) was significantly lower than that of the negative control group (2.775±0.094; Table I: F=269.57, P<0.05). The OPN level in the tumor tissues of the late intervention group (1.898±0.342) was also significantly lower than that of the negative control group (2.775±0.094; Table II: F=73.318, P<0.05) and the OPN level in the tumor tissues of the late intervention group (1.898±0.342) was significantly lower than that of the early intervention group (1.079±0.345; Table III: F=34.12, P<0.05). In the negative control group without thalidomide treatment, OPN (2.775±0.094) was highly expressed in the hepatocellular carcinoma tissues and was at a significantly higher level than that in the pericarcinomatous tissues (Table IV: F=328.74, P<0.05).

## Discussion

OPN is a secretory phosphorylated glycoprotein, with a relative molecular mass of ∼44 kDa, containing ∼300 amino acid residues, of which aspartic acid, serine and glutamic acid residues account for a high proportion. It has been demonstrated that OPN is largely synthesized and secreted in malignant tumor cells, particularly in hepatocellular carcinoma (1,2). The intramolecular structure, RGD (Arg-Gly-Asp), is an unique sequence that improves cell adhesion in proteins. Through the RGD cell adhesion sequence, OPN may interact with important tumor metastasis factors, including integrin, CD44, vascular endothelial growth factor/epidermal growth factor receptor (VEGF/EGFR), matrix metalloproteinases (MMPs), fibronectin (FN), survivin, transforming growth factor (TGF), tumor necrosis factor (TNF) and urokinase-type plasminogen activator (uPA) to promote cell chemotaxis, adhesion and migration (8–17). Budhu *et al*(18) and Pan *et al*(19) put forward the theory that OPN was a significant factor in hepatocellular carcinoma metastasis and that it may be a molecular marker of intrahepatic metastasis. These authors also suggested that OPN may act via the PI3K/NF-Kβ cell signaling pathway. In our preliminary studies (3,20,21), the excessive expression of OPN was was identifed to be closely correlated with the early metastasis and relapse of hepatocellular carcinoma, which is a significant factor in a poor prognosis following hepatectomy. OPN had varying expression levels in hepatoma cells with different metastatic potentials and was not expressed in normal hepatic cells. These differences were statistically significant. OPN was confirmed to have utility as a sensitive index for predicting micro-metastases in early hepatocellular carcinoma. OPN may be considered as a bridge connecting primary and metastatic tumors in hepatocellular carcinoma.

It has been reported (22) that thalidomide prevents basic fibroblast growth factor (bFGF) and VEGF from inducing angiogenesis, which may involve multiple pathways. However, the specific mechanism by which thalidomide inhibits angiogenesis remains unclear. We have previously attempted to use thalidomide intervention in 7 patients with unexplained, permanent hematochezia in the clinic and observed unexpected effects. We speculated that thalidomide may be related to uPA (23). Thalidomide may block the blood flow to malignant tumors and the resultant lack of nutrition may then reduce the growth of tumor cells and cause them to atrophy, thereby extending the lives of affected patients. Matrix metalloproteinase-9 (MMP-9) is a significant member of the MMP family, with the largest molecular weight (92 kDa). MMP-9 decomposes the extracellular matrix (ECM) and is involved in a number of physiological and pathological processes in the human body. Collagen types IV, V, VII and X, gelatins and elastin fibers are its main substrates. MMPs degenerate the basement membrane barrier and act with various cytokines to promote the formation of new blood vessels in tumors and the proliferation of tumor cells (24). MMPs may also participate in evasion of the host’s immune surveillance to promote the growth of tumors, as well as participating in invasion and metastasis. We speculate that thalidomide may affect the activity and expression of VEGF to reduce the stimulation of endothelial cells which produce MMP-9. Alternatively, thalidomide may regulate the balance of MMP-9 and its inhibitors, the tissue inhibitors of MMPs (TIMPs), to attenuate the cascading matrix degradation process and affect the activities of MMP-9 and other proteases which aid the degeneration of the basement membrane and ECM. Reduced vascular permeability may increase the resistance of MHCC97 cells to penetration, which would also affect the migration of endothelial cells and constrain the angiogenesis, infiltration and metastasis of MHCC97. However, the specific mechanism of action remains unknown. OPN is highly expressed in most malignant tumors, has been shown to be an important tumor metastasis factor and is considered the molecular trigger of invasion and metastasis of hepatocellular carcinoma due to its teratogenic effects. Thalidomide was abandoned for numerous years. In recent years, due to its clinical treatment for certain tumors and obvious therapeutic effect, thalidomide has gained support, but the mechanism of tumor inhibition is unclear and widespread clinical application is restricted.This study selected OPN as a main target and showed that thalidomide is able to damage human hepatocellular carcinoma cells and downregulate the expression of osteopontin. The present study has certain scientific significance and potential social significance. Uncertainty concerning whether thalidomide is correlated with OPN and the nature of the correlation remains. In the present study, thalidomide was applied to modulate the expression of OPN in hepatocellular carcinoma tissues and the results revealed predictable effects which indicated that OPN may be one of the targets of thalidomide. We observed the encouraging appearance that thalidomide have the imaging efforts, but the deeper mechanism remains unknown. We suggest that thalidomide may block the vascularization of the cancer and destroy the circle or cancer cell. Although no literature has previously reported studies of thalidomide and OPN, it is likely that there is a inner correlation between them.

Certain scholars (25) believe that in order to clarify the molecular pharmacological mechanism of thalidomide, emphasis should be placed on the upstream molecules of NF-κB, including searching for a specific thalidomide-binding factor to inhibit the phosphorylation of NF-κB. NF-κB is a protein factor, which specifically combines with the enhancer κB sequence of the κ-light chain gene in immunoglobulin located in extracts of B-cell nuclei. NF-κB has been identified to play a significant role in the occurrence and development of a number of diseases. The anti-apoptotic and immune activation functions of NF-κB and its ability to promote cell proliferation are potentially factors that lead to normal cells becoming malignant. Thalidomide may attenuate the phosphorylation process induced by TNF-α and other factors. This would inhibit the proteins from separating from NF-κB and prevent NF-κB from passing into the nucleus, thereby resulting in immune adjustment and an anti-angiogenic effect. The authors identified that thalidomide had a potential inhibitory effect on the NF-κB signaling pathway. A mouse model of hepatic cirrhosis was established and research was conducted into the effect of thalidomide on the expression of NF-κB, IKB, inter-cellular adhesion molecule-I (ICAM-I) and vascular CAM-1 (VCAM-1). Thalidomide was shown to markedly decrease the expression of NF-κB, ICAM-I and VCAM-1. Whether OPN was involved in these processes and how it contributed to them require further study.

In conclusion, as an older drug with a mature production line, thalidomide is cheap and convenient to use. The specific mechanism of thalidomide requires clarification if it is intended to be comprehensively used as a clinical antitumor drug or to replace or be used in combination with expensive new drugs. This would assist in correcting the historical prejudices against thalidomide. Previous studies into thalidomide have shown that it plays a significant role in the treatment of various difficult and severe diseases. With the continuous development of clinical and pharmacological studies, the effects of thalidomide and its mechanism of action may become clearer and better defined. The present study supports the positive effect of thalidomide as an anticancer tratment that is cheap. The present results show that tholidomide prohibits the liver cancer as it targets osteopontin.

## Figures and Tables

**Table I:** Analysis of variance of the value of immunohisto-chemistry in the early intervention and negative control groups.

Group	Sum of squares	Degree of freedom	Square of mean	F-value	P-value
Inter	17.26	1	17.255		
Intra	1.408	22	0.064	269.57	0.0091
Total	18.66	23			

Inter, between the early intervention and negative control groups; Intra, within the early intervention and negative control groups; Total, the sum of the early intervention and negative control groups.

**Table II:** Analysis of variance of the value of immunohistochemistry in the late intervention and negative control groups.

Group	Sum of squares	Square of mean	F-value	P-value
Inter	4.611	4.611		
Intra	1.384	0.063	73.318	0.0184
Total	5.995			

Inter, between the late intervention and negative control groups; Intra, within the late intervention and negative control groups; Total, the sum of the late intervention and negative control groups.

**Table III:** Results of analysis of variance of the value of immunohistochemistry in the early intervention and late intervention groups.

Group	Sum of squares	Degree of freedom	Square of mean	F-value	P-value
Inter	4.026	1	4.026		
Intra	2.596	22	0.118	34.12	0.0372
Total	6.622	23			

Inter, between the early intervention and late intervention groups; Intra, within the early intervention and late intervention groups; Total, the sum of the early intervention and late intervention groups.

**Table IV:** Analysis of variance of the value of immunohisto-chemistry in the tumor tissues and pericarcinomatous tissue of the negative control group.

Group	Sum of squares	Degree of freedom	Square of mean	F-value	P-value
Inter	9.767	1	9.767		
Intra	0.654	22	0.03	328.74	0.0064
Total	10.42	23			

Inter, between the tumor tissues and pericarcinomatous tissue of the negative control group; Intra, within the tumor tissues and pericarcinomatous tissue of the negative control group; Total, the sum of the tumour tissue and pericarcinomatous tissue groups.
